# SARS-CoV-2 and Skin: The Pathologist’s Point of View

**DOI:** 10.3390/biom11060838

**Published:** 2021-06-04

**Authors:** Gerardo Cazzato, Giulia Mazzia, Antonietta Cimmino, Anna Colagrande, Sara Sablone, Teresa Lettini, Roberta Rossi, Nadia Santarella, Rossella Elia, Eleonora Nacchiero, Michele Maruccia, Andrea Marzullo, Eugenio Maiorano, Giuseppe Giudice, Giuseppe Ingravallo, Leonardo Resta

**Affiliations:** 1 Department of Emergency and Organ Transplantation (DETO), Section of Pathology, University of Bari “Aldo Moro”, 70124 Bari, Italy; giulia.mazzia1@gmail.com (G.M.); micasucci@inwind.it (A.C.); anna.colagrande@gmail.com (A.C.); lettinit@yahoo.com (T.L.); roberta.rossi@policlinico.ba.it (R.R.); nadia.santarella@policlinico.ba.it (N.S.); andrea.marzullo@uniba.it (A.M.); eugenio.maiorano@uniba.it (E.M.); giuseppe.ingravallo@uniba.it (G.I.); leonardo.resta@uniba.it (L.R.); 2 Department of Interdisciplinary Medicine, Section of Legal Medicine, Policlinico di Bari Hospital, University of Bari, 70124 Bari, Italy; sarasabloneml@gmail.com (S.S.); giuseppe.giudice@uniba.it (G.G.); 3 Department of Emergency and Organ Transplantation, Section of Plastic and Reconstructive Surgery, University of Bari, 70124 Bari, Italy; rossellaelia4@gmail.com (R.E.); eleonora.nacchiero@yahoo.it (E.N.); michele.maruccia@uniba.it (M.M.)

**Keywords:** SARS-CoV-2, COVID-19, skin, eruption

## Abstract

The SARS-CoV-2 pandemic has dramatically changed our lives and habits. In just a few months, the most advanced and efficient health systems in the world have been overwhelmed by an infectious disease that has caused 3.26 million deaths and more than 156 million cases worldwide. Although the lung is the most frequently affected organ, the skin has also resulted in being a target body district, so much so as to suggest it may be a real “sentinel” of COVID-19 disease. Here we present 17 cases of skin manifestations studied and analyzed in recent months in our Department; immunohistochemical investigations were carried out on samples for the S1 spike-protein of SARS-CoV-2, as well as electron microscopy investigations showing evidence of virions within the constituent cells of the eccrine sweat glands and the endothelium of small blood vessels. Finally, we conduct a brief review of the COVID-related skin manifestations, confirmed by immunohistochemistry and/or electron microscopy, described in the literature.

## 1. Introduction

At the end of December 2019, a new Coronavirus strain was initially reported in Wuhan, China, Hubei Province [1]. Within a few months, the largest and most advanced health systems in the world would be faced with a pandemic capable of determining globally, as of 5:15 p.m. CEST, 10 May 2021, 157,973,438 confirmed cases, with 3,288,455 deaths [2]. Although, like most of the previous respiratory viruses (MERS and SARS-CoV-1) [3,4], the lung was the organ most frequently affected by the pathological manifestations, it was soon clear that other organs could also be affected by SARS-CoV-2. Among these, the skin has been the subject of great interest, so much so that it has been hypothesized that it could be a real “sentinel” of early COVID-19 manifestations. Over the months, the clinical patterns described as correlated to SARS-CoV-2 and the different histopathological patterns were so different and varied as to make it possible to distinguish them in different classifications. During these months, we have had the opportunity to study a series of 17 cases of COVID-positive patients; on skin biopsies, we made the opportune immunohistochemical (IHC) and molecular (PCR) analyses and electron microscopy evaluation for confirmatory purposes. A brief review is then made of the Italian literature in which cases of COVID-related skin manifestations included data on immunohistochemical and/or electron microscopy techniques.

## 2. Materials and Methods

We pooled the skin biopsy samples received at our Pathological Anatomy Laboratory between 4 April 2020 and 10 May 2021. All patients had undergone molecular swab tests for SARS-CoV-2 by GeneXpert Dx Xpress SARS-CoV-2 RT-PCR (Cepheid) [5]. The analytical sensitivity and specificity of this test are reported by the manufacturers as 100% (87/87 samples) and 100% (30/30 samples), respectively, with a detection limit of 250 copies/mL or 0.0100 plaque-forming units per milliliter [6]. All biopsies were performed as punch biopsy (4/6 mm), and the samples were fixed in buffered formaldehyde at 10%, sampled according to guidelines, processed, paraffin-embedded, microtome-cut (5-micron thickness) and stained with routine Hematoxylin/Eosin staining (H & E). They were observed with an Olympus BX-51 Optical Microscope (Shinjuku Monolith, 2-3-1 Nishi-Shinjuku, Shinjuku-ku, Tokyo, Japan) equipped with the Olympus DP80 image acquisition system. On all samples, IHC investigations were performed with anti-SARS-CoV-2 spike S1 glycoprotein monoclonal antibody, Thermofisher, Rabbit, at pH 6, diluted 1:800, and antigenic unmasking heat-induced citrate buffer epitope retrieval, for enzymatic IHC analysis. Lung sections of COVID subjects were used as positive controls and lungs of swab negative subjects as negative controls. In addition, electronic microscopy analysis was performed: at the time of biopsy, a very small skin sample was immediately fixed in 2.5% Gluteraldehyde for 4 h at 4 °C, and after overnight immersion in phosphate buffer, post-fixed with Osmium Tetroxide in PBS for 2 h at a temperature of 4 °C. The prepared samples were processed for inclusion in araldite epoxy resin (M) CY212 (TAAB, Aldermason, UK). Semithin sections 0.5 µm thick were stained with Toluidine Blue for microscopic analysis. Ultrathin sections were mounted on nickel grilles with uranium acetate and lead citrate contrast. The semithin sections were observed with a Nikon photomicroscope equipped with a Nikon Coolpix DS-U1 Digital Camera (Nikon Instruments SpA, Calenzano, Italy). The ultrathin sections were observed with a Morgagni 268 electron transmission microscope (FEI Company, Naples, Italy). All skin biopsies were positive for molecular investigations performed by Polymerase Chain Reaction (PCR), with a viral genome copy number ranging between medium and low.

All cases were examined independently under double-blind conditions by two pathologists with expertise in the dermatopathology field, in order to confirm the diagnoses.

Finally, we conduct a brief review of the current literature on cases of patients with a positive SARS-CoV-2 swab who underwent skin biopsy investigated by immunohistochemistry and/or electron microscopy. We performed this review using Pubmed and Web of Science as search engines, applying the following key words: “COVID-19” OR “n-CoV 19” OR SARS-CoV-2 and “skin “OR” skin lesions “OR” skin manifestation “OR” COVID-toes “and” immunohistochemistry “OR/AND” electron microscopy “.

## 3. Results

We subdivided the patients presented in our study according to clinical-histopathological characteristics in order to derive four different groups.

### 3.1. Erythema-Multiforme Like Eruptions

**Figure 1:**
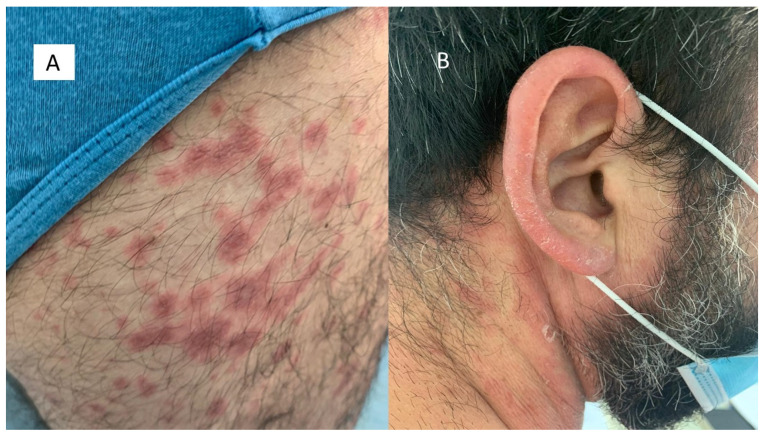
(**A**) Examples of an erythema multiforme-like lesion in the first group of patients. Note the detail of the violaceous erythematous well-defined border lesions with a typical targetoid aspects; (**B**) “Targetoid” type lesions with initial desquamation at the level of the periauricular area.

**Figure 2:**
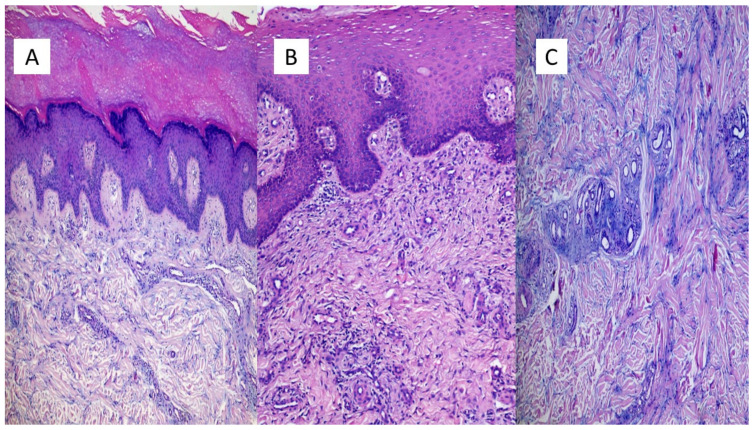
(**A**) Example of an erythema-multiforme like lesion of the first group of patients. In (**A**), we note the presence of modest ortho-parakeratosis (sampling from the pre-auricular area), with acanthosis and a mild inflammatory lymphomonocytic infiltrate (Hematoxylin-Eosin, Original Magnification: 4×. (**B**) Histological examination revealed a modest inflammatory infiltrate mainly peri-vasally, in the superficial and middle dermis (sample taken from the back) (Hematoxylin-Eosin, Original Magnification: 10×); (**C**) Histological details of the dermal edema and mucinosis of the hair appendages (Hematoxylin-Eosin, Original Magnification: 20×).

**Figure 3:**
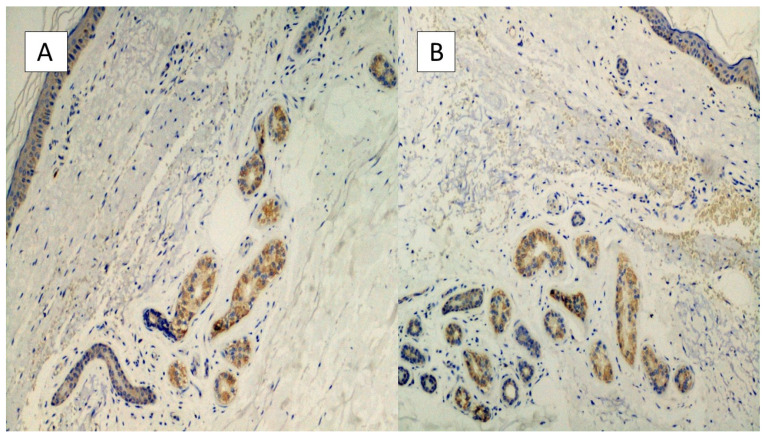
(**A**,**B**) Histological preparation for immunostaining with Anti-SARS-CoV-2 monoclonal antibody. Note the granular, cytoplasmic positivity at the level of the cells constituting the eccrine sweat glands. Involvement of the vascular endothelium is rarely described (IHC, Original Magnification: 10×).

**Figure 4:**
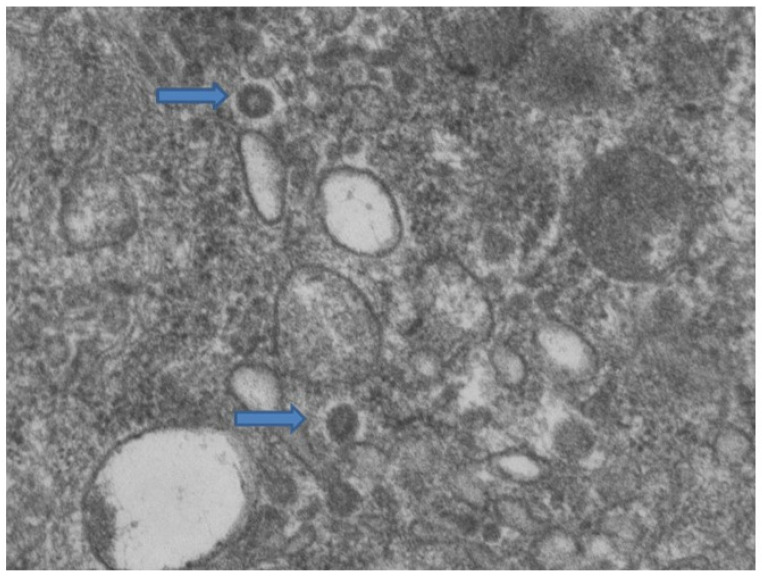
Cytoplasm of eccrine sweat glands. Among the cellular organelles (mitochondria and endoplasmic reticulum) the presence of two virions is observed, indicated by arrows (Electron microscopy, Original Magnification: 44,000×).

The first group included seven patients aged between 39 and 64 who had developed erythema-multiforme skin manifestations, with erythematous-edematous papules mainly located on the trunk and limbs, and less frequently on the extremities, that progressively turned into erythemato-violaceous patches with a dusky center, showing the appearance of typical target lesions. An example of such manifestations is shown in Figure 1A,B. In all seven cases, patients developed skin manifestations 3–8 days after the SARS-CoV-2 swab. Histopathological examination of the skin biopsies of this group of patients was quite comparable: the presence of modestly ortho-parakeratotic skin with epidermal apoptosis phenomena was described; modest vacuolization of the keratinocytes of the basal layer and a mild inflammatory infiltrate, mainly lymphomonocytic, within the superficial derma was associated with edema (Figure 2A–C). The IHC reactions carried out showed a strong positivity at the cytoplasmic level of the cells constituting the excretory portion of the eccrine sweat glands (Figure 3A,B) and, more rarely, positivity was observed in the endothelium of the small blood vessels. Based on the IHC indications, the sporadic presence of virions among the cell organelles was observed in the epithelial cells belonging to the eccrine sweat glands (Figure 4).

### 3.2. Pseudochildblains

**Figure 5:**
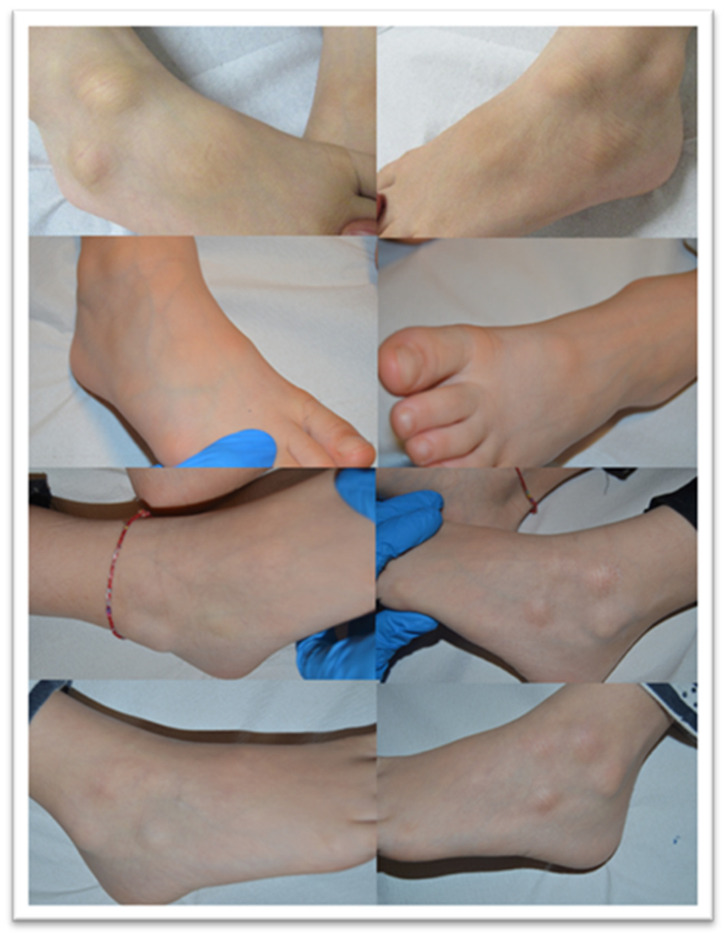
Clinical appearance of the lesions in the second group of patients studied, showing a nodular appearance and overlying erythematous skin.

**Figure 6:**
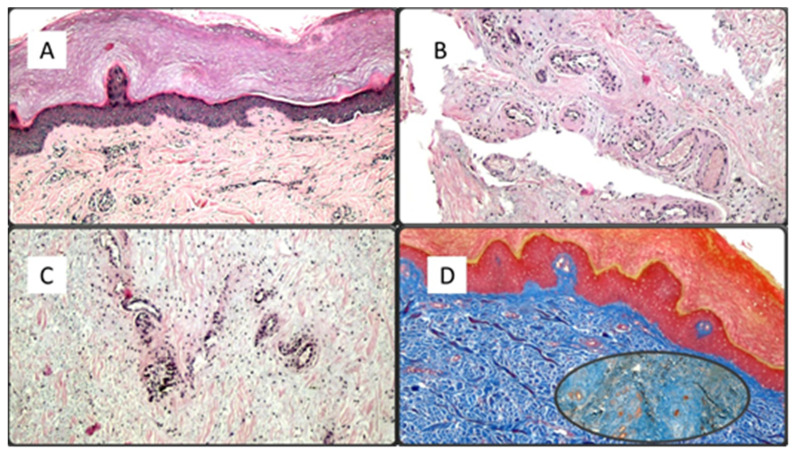
(**A**) Histological preparation demonstrating mild ortho-parakeratosis, focal vacuolation of the keratinocytes of the basal layer and mild inflammatory infiltration of the superficial dermis (Hematoxylin-Eosin, 4×); (**B**,**C**) Histological details of the superficial capillary plexus vessels characterized by swollen endothelial cells, of the “hobnail” type, with a narrow lumen and focal thrombotic phenomena (Hematoxylin-Eosin, 10× and 40×); (**D**) Masson’s trichrome stain which highlights the preservation of collagen fibers in the superficial dermis and their fragmentation at the level of the medial/deep dermis (Masson’s Trichrome stain, Original Magnification: 10×, box: 20×).

**Figure 7:**
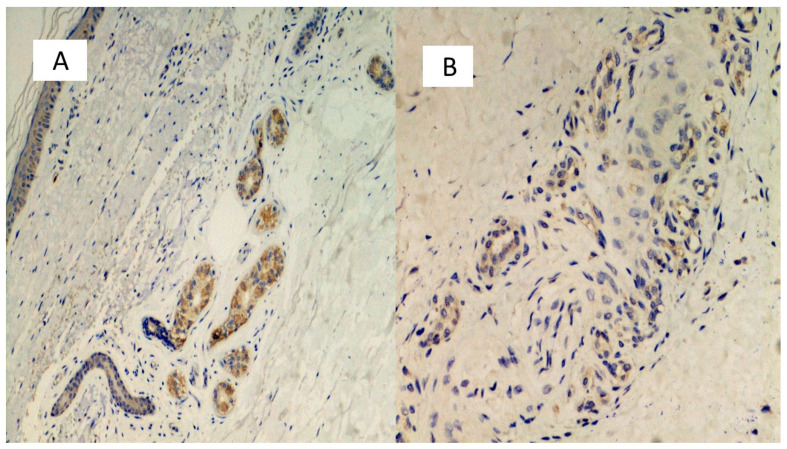
(**A**,**B**) Preparations for immunostaining with anti-SARS-CoV-2 antibody, showing positivity in the eccrine sweat glands cells, but also in the vascular endothelium (**B**).

**Figure 8:**
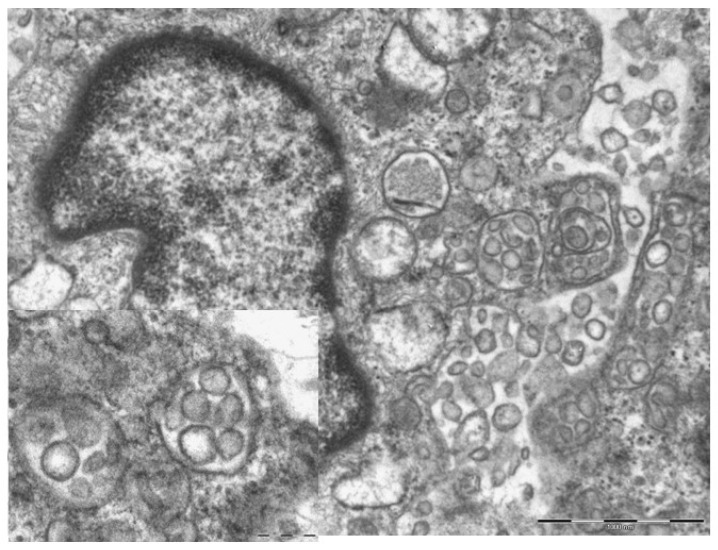
The photomicrograph shows the endothelial cell cytoplasm, with numerous vacuoles within which viral particles are observed (14,000 x and 71,000 box).

Another seven patients studied in these months were classified in a second group in relation to their clinical-pathological skin manifestations. In particular, these are mostly adolescent patients, aged between 12 and 23, who had developed acro-localized lesions during the COVID-19 pandemic. All seven patients had undergone molecular swab tests for SARS-CoV-2 which documented positivity to the virus. Clinically, the lesions were nodular, bilateral almost symmetrical, localized at the level of the malleolar and peri-malleolar regions. The overlying skin was slightly erythematous, without further alterations. From the clinical point of view, these cutaneous manifestations resembled Granuloma annulare and/or erythema elevatum diutinum (Figure 5). Histological examination revealed focal vacuolation of the keratinocytes of the basal layer (Figure 6A), with almost complete preservation of the collagen fibers at the level of the superficial dermis but reduction up to complete disappearance at the level of the medium-deep dermis (Masson’s trichrome stain, (Figure 6D and box)). Small blood vessels belonging to the superficial capillary plexus feature a narrow lumen, prominent “Hobnail” endothelial elements, extensive edema and thrombotic phenomena (Figure 6B,C). Also in this second group of patients, all biopsies examined with Anti-SARS-CoV-2 monoclonal antibody were positive, most evidently at the level of the eccrine sweat glands cytoplasm but also of the vascular endothelium (Figure 7A,B). The endothelial cells showed, in some cases, the presence of coarse cytoplasmic vesicles in which virions were identifiable (Figure 8).

### 3.3. Chickenpox Rush

Two patients belonging to the third group developed a varicella-like exanthem, a few days after a molecular swab ascertained positivity to SARS-CoV-2. Patients were 38 and 42 years old and presented with a rash that turned into itchy, fluid-filled blisters forming scabs. The rash affected the chest, back, and face, and, in the second case, the entire skin surface. Histological examination revealed vacuolar degeneration of the basal layer with multinucleate, hyperchromatic keratinocytes and dyskeratotic cells. There was no significant inflammatory infiltrate. Also in these two cases, the IHC reactions were positive, featuring the same pattern as the previous ones.

### 3.4. Urticarioid Rash

A 43-year-old patient presented with a smooth urticarial rash, with slightly elevated papules that were erythematous and provoked severe pruritus. Histologically, dermal edema was present, with sparing of the epidermis; a moderate perivascular inflammatory infiltrate was present, with eosinophils and dilated blood vessels. In addition, in this last case, IHC for SARS-CoV-2 was positive. Table 1 summarizes the clinical-pathological characteristics of our series.

## 4. Discussion

The SARS-CoV-2 pandemic has affected the entire world, and in a few months numerous different scientific works have highlighted how many possible manifestations there can be [7]. In particular, although the lung was the most commonly affected organ, it was soon clear that the skin could also be affected, with different patterns [8]. We have published a previous work [9] describing cases of skin manifestations that occurred in the first months of 2020, at the dawn of the SARS-CoV-2 pandemic. In this work, we present new cases that we had the opportunity to study during the second pandemic wave and characterized by means of immunohistochemical and electron microscopy investigations. In the literature, there are few articles relating to skin biopsies on which such investigations have been carried out. In a recent review, Rongioletti et al. [10] presented polymorphic aspects that can be observed on the skin of SARS-CoV-2 positive subjects. In particular, the authors investigate the clinical evidence that patients who develop Pseudochilblain lesions tend to have a better disease course, compared to patients with acro-ischemic/livedoid necrotic lesions, who tend to have a worse clinical outcome related to a state of impairment of the coagulation system. The same authors highlight the importance of a correct execution of immunohistochemical investigations and electron microscopy, especially in the presence of negative molecular data (PCR). Almost all of the cases analyzed by IHC and/or electron microscopy belong to pediatric subjects, like those we describe. In detail, Colmenero et al. [11] described the cases of seven children with chilblains, all negative for the molecular test for SARS-CoV-2 and with a similar histopathological picture to that described in this work. Immunohistochemical investigations conducted on all biopsies were positive. In two cases, it was possible to perform ultrastructural study, in one of which they described viral particles attributable to the virus. Torrelo et al. [12] described four children with chilblains and erythema multiforme-like skin manifestations. Of these, one child was positive to the molecular swab, while the others were negative. A biopsy was performed on the erythematous lesions of all patients, and two of these had atypical histopathological features, but all were IHC positive for SARS-CoV-2. Hachem et al. [13] described 19 cases of pediatric patients, all negative for SARS-CoV-2 molecular swabs, on which biopsies with histological features similar to those we describe were performed in the second group of patients. Ultrastructural study was performed on four cases, none of which were considered positive, but non-specific lesions were found. Andina et al. [14] also described a case of a one-month-old baby girl with reticulated plantar purpura, negative to the molecular swab, but positive to IHC for SARS-CoV-2. We also, in another very recent work [15], described three cases of molecular test negative pediatric patients whose skin biopsies showed histological characteristics similar to those described in this new study, in the second group, who had tested positive for IHC for SARS-CoV-2.

## 5. Conclusions

We have described these new cases which are added to others previously described even if rarely accompanied by immunohistochemical investigations and electron microscopy. It should, however, be noted that the electron microscopy findings are suggestive but not conclusive with absolute certainty regarding the virion.

Over time, an expansion of the series and cases published in Literature will probably contribute to elucidating on the one hand the polymorphic aspects of skin manifestations from SARS-CoV-2 and on the other hand help to clarify the etiopathogenetic mechanisms.

## Figures and Tables

**Table 1:** Summary of skin manifestations found in our study.

Skin Manifestation	Number of Patients	Age (Years)	Localization	Immunohistochemistry	Electron Microscopy
Erythema Multiforme-Like	7	39–64	Widespread	Positive	Positive
Pseudochildblains	7	12–23	Foot and malleolar region	Positive	Positive
Chickenpox rash	2	38,42	Chest, back, and face	Positive	Not carried out
Urticarioid rash	1	43	Widespread	Positive	Not carried out
